# Rapid Determination of Macrolide and Lincosamide Resistance in Group B Streptococcus Isolated from Vaginal-Rectal Swabs

**DOI:** 10.1155/2007/46581

**Published:** 2007-06-26

**Authors:** Wilfred P. Dela Cruz, Joann Y. Richardson, Judith M. Broestler, Jennifer A. Thornton, Patrick J. Danaher

**Affiliations:** ^1^Clinical Investigation Facility, David Grant USAF Medical Center, Travis Air Force Base, CA 94535, USA; ^2^Department of Pediatrics, F. Edward Hébert School of Medicine, The Uniformed Services University of the Health Sciences, Bethesda, MD 20814-4799, USA; ^3^Medical Laboratory Flight/Microbiology Department, David Grant USAF Medical Center, Travis Air Force Base, CA 94535, USA; ^4^Department of Infectious Diseases, Eglin USAF Regional Hospital, Eglin Air Force Base, FL 32542-1282, USA

## Abstract

*Objective*. Our objective was to assess the ability of real-time PCR to predict in vitro resistance in isolates of group B streptococcus (GBS). *Methods*. The first real-time PCR assays for the genes known to confer resistance to erythromycin and clindamycin in GBS were developed. Three hundred and forty clinical GBS isolates were assessed with these assays and compared with conventional disk diffusion. *Results*. The presence of an erythromycin ribosome methylation gene (*erm*B or *erm*TR variant A) predicted in vitro constitutive or inducible resistance to clindamycin with a sensitivity of 93% (95% CI 86%–97%), specificity of 90% (95% CI 85%–93%), positive predictive value of 76% (95% CI 67%–84%), and negative predictive value of 97% (95% CI 94%–99%). *Conclusion*. This rapid and simple assay can predict in vitro susceptibility to clindamycin within two hours of isolation as opposed to 18–24 hours via disk diffusion. The assay might also be used to screen large numbers of batched isolates to establish the prevalence of resistance in a given area.

## 1. INTRODUCTION

Each year in the US 8000 neonatal cases of sepsis due to GBS are reported [1]. Up to 30% of women carry GBS in the urogenital tract, intestinal tract, or both. Intrapartum antibiotic chemoprophylaxis remains the key to preventing neonatal disease. Penicillin is the drug of choice for prophylaxis and treatment of GBS infection. In patients allergic to penicillin, erythromycin and clindamycin are the commonly used alternatives. The proportions of GBS isolates with in vitro resistance to erythromycin and clindamycin have steadily increased since 1996 [2]. The prevalence of resistance among invasive GBS isolates in the United States and Canada ranged from 7% to 25% for erythromycin and from 3% to 15% for clindamycin in reports published between 1998 and 2001.

The genetic basis for macrolide and lincosamide resistance in GBS has been studied extensively [3]. The *erm*TR variant A (*erm*TR) and *erm*B genes modify a site in 23S rRNA common to the binding of macrolides, lincosamides, and streptogramin B antibiotics. The macrolide efflux (*mef*A) gene allows the bacterium to actively expel the antibiotic from the interior of the cell [4]. Both the *erm*TR and *erm*B genes have been associated with the MLS_B_ constitutive phenotype in GBS (erythromycin-resistant, clindamycinresistant); the *erm*TR gene with the MLS_B_ inducible phenotype (erythromycin-resistant, clindamycin-susceptible, D-test positive); and the *mef*A gene with the M phenotype (erythromycin-resistant, clindamycin-susceptible, D-test negative) [5].

Current recommendations state that all pregnant women should be screened at 35–37-week gestation for vaginal and rectal GBS colonization, and that susceptibility testing should be performed on isolates from women with penicillin allergy [2]. The CDC further recommends D-testing for GBS isolates that are erythromycin-resistant and clindamycinsusceptible on initial disk diffusion testing [6]. Vancomycin is now the recommended antimicrobial prophylaxis for perinatal GBS disease prevention in the penicillin allergic patient whose isolate is known to be resistant to erythromycin and clindamycin (constitutively or inducibly), or whose isolate has not been tested for resistance. This policy is at odds with Hospital Infection Control Practices Advisory Committee (HICPAC) guidelines urging limitation of vancomycin use [7].

While a newly licensed PCR-based diagnostic test [8] allows for intrapartum detection of GBS colonization in women who have had no prenatal care or for whom culture results are otherwise unknown, vancomycin would still be indicated in the penicillin allergic patient with a positive test result due to a lack of susceptibility information. The disk diffusion method, which takes 18–24 hours to be performed, is currently the most commonly used method for determining GBS susceptibilities. An assay for the rapid detection of the *erm* and *mef*A genes might allow for more judicious use of vancomycin. The aim of the present study was to develop rapid assays using real-time PCR for detection of these genes and to assess their performance using the disk diffusion method as the standard. A secondary goal was to assess the susceptibility of isolates demonstrating constitutive clindamycin resistance to a new ketolide antibiotic, telithromycin.

## 2. METHODS

Three hundred and forty consecutive, unique patient GBS isolates from screening cultures collected at our facility in 2004 were studied. Disk diffusion testing of the isolates was accomplished according to National Committee for Clinical Laboratory Standards (NCCLS) guidelines [9]. Briefly, GBS growth from an overnight (18-hour) sheep blood agar plate was suspended in 0.9% saline to a density equivalent to the turbidity of the 0.5 McFarland standards. This suspension was inoculated on Mueller-Hinton agar supplemented with 5% defibrinated sheep blood. Erythromycin (15 *μ*g, Remel, Lenexa, Kan, USA) and clindamycin (2 *μ*g, Remel) disks were placed on the plates. The plates were incubated at 35°C in an atmosphere of 5% CO_2_ for 20 to 24 hours beforemeasuring zones of inhibition.

D-testing was performed for erythromycin-resistant/clindamycin-susceptible isolates, as described previously [10]. Clindamycin disks and erythromycin disks were placed approximately 15mm apart on Mueller-Hinton agar supplemented with 5% defibrinated sheep blood that had been inoculated with a standardized (0.5 McFarland) suspension of GBS. The plates were incubated at 35°C in an atmosphere of 5% CO_2_ for 20 to 24 hours before observing for D-shaped blunting of the circular zone of inhibition around the clindamycin disks on the side facing the erythromycin disk. Further susceptibility testing was performed for 50 isolates exhibiting an MLS_B_ constitutive phenotype using telithromycin (15 *μ*g, Sanofi-Aventis, Bridgewater, NJ, USA) disks.

Genomic DNA from GBS isolates was extracted using the MagNA Pure LC automated extraction system (Roche Applied Science, Indianapolis, Ind, USA) per manufacturer's recommendation. Real-time fluorescent PCR was used to detect *cfb*, a GBS-specific gene target, and the MLS resistance genes (*erm*TR, *erm*B and *mef*A). Discordant results were retested for confirmation.

The primers and hybridization fluorescent resonance energy transfer (FRET) probes were designed using the LightCycler probe design software version 1.0 (Roche Applied Science, Indianapolis, Ind, USA). The primers for the 247 bp fragment of *cfb* gene (GenBank accession no. NC_004116) are as follows: forward primer (33–48) 5′ to 3′, AACTCTAGTGGCTGGT; and reverse primer (279–264) antisense 5′ to 3′, GGCACGCAATGAAGTC. The internal FRET hybridization probes for *cfb* gene fragment have the following sequence: upstream probe (93–112) 5′ to 3′, AGTGACAACTCCACAAGTGG-FITC; downstream probe (115–144) 5′ to 3′, 640RED-AATCATGTAAACAGTAATAATCAAGCCCAG-PHOSPHATE. The primers for the 162 bp fragment of *erm*B (GenBank accession no. X72021) are as follows: forward primer (390–406) 5′ to 3′, CTACAAGCGTACCTTGG; and reverse primer (551–533) antisense 5′ to 3′, TCTGGAACATCTGTGGTAT. The internal FRET hybridization probes for 162 bp *erm*B gene fragment have the following sequence: upstream probe (468–483) 5′ to 3′, GCTGCCAGCGGAATGC-FITC; downstream probe (486–5143) 5′ to 3′, 640RED-TCATCCTAAACCAAAAGTAAACAGTGTCT-PHOSPHATE. The primers for the 281 bp fragment of *erm*TR gene (GenBank accession no. AF443782) are as follows: forward primer (505–524) 5′ to 3′, CCTTATTGTAGAGAGGGGAT; and reverse primer (785–768) antisense 5′ to 3′, GCTTCAGCACCTGTCTTA. The internal FRET hybridization probes for *erm*TR gene detection are the following: upstream probe (607–627) 5′ to 3′, GCCACGAGCATATTTTCACCC-FITC; downstream probe (630–660) 5′ to 3′: 640RED-AGCCTAATGTAGATTCTGTATTGATTGTACT-PHOSPHATE-. The primers for the 179 bp fragment of *mef*A gene (GenBank accession no. AY071836) are as follows: forward primer (294–309) 5′ to 3′, GGAGCTACCTGTCTGG; and reverse primer (472–457) antisense 5′ to 3′, CAACTGCCGGACTAAC. The internal FRET hybridization probes for *mef*A gene detection are the following: upstream probe (341–364) 5′ to 3′, TTGGAACAGCTTTTCATACCCCAGFITC; downstream probe (367–388) 5′ to 3′, 640RED-CTCAATGCGGTTACACCACTTT-PHOSPHATE. The realtime fluorescent PCR cycling was carried out in the LightCycler (Roche Applied Science, Indianapolis, Ind, USA) in 20 *μ*L reaction mixture containing 5 *μ*L (∼1 ng) template, 5 mM MgCl_2_, 0.2 mM dNTP, 1 U Taq polymerase, 500 nM forward and reverse primers, and 100 nM upstream and downstream hybridization probes. Cycling conditions include an initial denaturation step at 94°C for 60 seconds, followed by 35 cycles which consist of annealing at 60°C for 30 seconds, extension at 72°C for 5 seconds, and denaturation at 94°C for 1 second. Progress of real-time fluorescent PCR was monitored in channel 2 on the LightCycler. During the optimization phase of the real-time PCR assays, the production of a single PCR product was confirmed in 3% agarose gel electrophoresis visualized by SYBR Green (Molecular Probes, Eugene, Ore, USA) staining at 1:10 000 dilution.

STATA version 9.0 (College Station, Tex, USA) was used to calculate sensitivity, specificity, positive predictive value, and negative predictive value, and their corresponding 95% confidence intervals.

## 3. RESULTS

**Figure 1:**
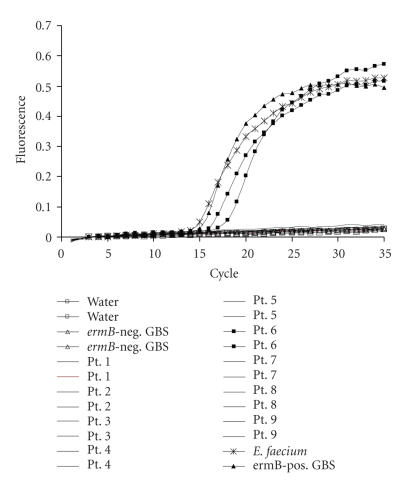
The intensity of the fluorescence signal (F2) versus cycle number plot. A 162 bp fragment of *erm*B gene was amplified from genomic DNA of *E. faecium* (asterisk), *erm*B-positive GBS isolate (closed triangle), and GBS isolated from patient number 6 (closed square). No fluorescence amplification was observed in *erm*B-negative GBS (ATCC 12386) (open triangle) and negative control (water, open square), or the rest of patient isolates tested.

**Figure 2:**
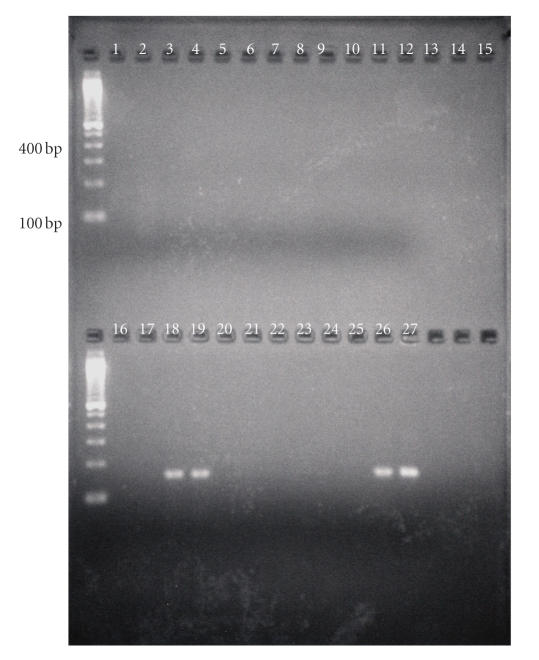
Agarose gel electrophoresis. PCR amplification products in *E. faecium*, *erm*B-positive GBS, and isolate from patient number 6 were confirmed with 3% agarose gel electrophoresis. Lanes 1-2 are PCR H2O, Lanes 18-19 are patient number 6, Lane 26 is *E. faecium*, and Lane 27 is *erm*B-positive GBS control.

The PCR assays were optimized in the LightCycler instrument. Figures 1 and 2 demonstrate representative fluorescence curves and agarose gel electrophoresis results for the *erm*B assay. Each component assay was run in separate tubes with the same PCR reaction and cycling conditions.

A total of 340 unique clinical GBS isolates collected from vaginal-rectal swabs were analyzed in this study. All isolates were positive for GBS-specific *cfb* gene by real-time PCR. One hundred isolates (29%) demonstrated resistance to erythromycin and/or clindamycin via disk diffusion testing (Table 1). Among the 100 resistant isolates, 79 demonstrated an MLS_B_ constitutive phenotype, 8 an MLS_B_ inducible phenotype, and 11 an M phenotype, while 2 were susceptible to erythromycin, but were resistant to clindamycin. No resistance to telithromycin was detected among the 50 randomly selected MLS_B_ constitutive strains that were tested.

Among the 340 isolates, a single resistance gene was detected in 105 isolates (31%), while 21 isolates (6%) harbored two genes. The genotype and phenotype were discordant in 14% of isolates (Table 2). Among the 240 fully susceptible isolates, 33 (14%) contained at least one resistance gene. At least one resistance gene was detected in 93 of the 100 resistant isolates. Presence of the *mef*A gene predicted the M phenotype with a sensitivity of 55% (95% CI 23%–83%), specificity of 94% (95% CI 90%–96%), PPV of 22% (95% CI 9%–42%), and NPV of 98% (95% CI 96%–99%). Presence of at least one *erm* gene within an isolate predicted constitutive or inducible resistance to clindamycin with a sensitivity of 93% (95% CI 86%–97%), specificity of 90% (95% CI 85%–93%), positive predictive value of 76% (95% CI 67%–84%), and negative predictive value of 97% (95% CI 94%–99%).

Because we had no a priori estimates for negative predictive value, post hoc power analyses were conducted using the obtained NPV of 97% to assess whether the sample size collected was sufficient to exclude negative predictive values as low as 90% for *erm* gene predicting clindamycin resistance. In order to be 95% certain (power = 0.95) that a negative predictive value as low as 90% was excluded (alpha level, two-tailed = 0.05), at least 133 test negative patients would be necessary [11]. Our number of test negative patients exceeded this value, making the power more than adequate for our research question.

## 4. DISCUSSION

We developed a real-time PCR method that can be used to rapidly detect macrolide resistance genes (*erm*B, *erm*TR, and *mef*A) from a pure GBS isolate. Previous studies have focused on identifying genes present in resistant isolates of GBS. To make these molecular techniques useful to clinicians, one must ask the question in reverse: does the presence of one of these genes predict in vitro resistance and possible treatment failures? The critical issue is the disparity between genotype and phenotype. A previous study examined this relationship in 1043 strains of macrolide-resistant *Streptococcus pyogenes and Streptococcus pneumoniae* and found a 10.2% rate of error when predicting the genotype from phenotypic data [12]. While we observed a slightly higher level of discordance between genotype and phenotype in GBS (14%), our data suggest that the absence of detection of an *erm* gene predicts in vitro susceptibility to clindamycin with an NPV sufficient to make the assay clinically useful.

While this would cut the 18–24 hours that it takes to perform disk diffusion testing down to 1-2 hours, the ideal molecular test would be performed directly from a clinical specimen. Because many other bacteria that colonize the urogenital and intestinal tracts harbor *erm* genes, direct analysis of clinical specimens with these assays would likely not yield useful results. Moreover, *erm* genes are located in a conjugative *S. agalactiae* plasmid, pIP501, which is highly homologous to numerous plasmids found in other bacterial species [13]. Thus, detection of clindamycin resistance in GBS directly from a clinical specimen, as does a commercially available PCR-based test for detecting MRSA [14], may not be possible without prior isolation.

An estimated 3%–10% of the general population is allergic to penicillin [15]. It would be ideal if an alternate agent with 100% efficacy and with no propensity for inducing resistance was available. Telithromycin, the first ketolide to receive FDA approval in the US, may be such an agent. Our findings mirror those of other researchers: telithromycin is broadly active against GBS, including those strains exhibiting resistance to erythromycin and clindamycin [16, 17]. A recent report describing 51 telithromycin-susceptible strains of MLS_B_ constitutive GBS that showed inducible resistance to telithromycin in the presence of erythromycin does, however, raise the possibility that resistance might develop while a patient is on therapy and result in a treatment failure [18–20]. Still, given its excellent in vitro activity against this organism, the short course of therapy used for this indication, and the lack of convincing evidence regarding the clinical importance of this type of inducible resistance, clinical testing of telithromycin as intrapartum chemoprophylaxis against neonatal GBS infection seem warranted.

We observed two isolates that were erythromycin-susceptible and clindamycin-resistant. While altered expression or regulation of the *erm*TR gene present in one of these isolates might explain its phenotype, the other strain did not contain an *erm* or *mef*A gene. This phenotype, the genetic basis for which remains unknown, is rare in North America, but is already widespread in New Zealand [21]. The existence of such strains presents a pitfall in inferring clindamycin susceptibility from erythromycin susceptibility when only the latter is tested. This underscores the importance of performing in vitro testing for resistance to clindamycin on isolates obtained from persons with penicillin allergy.

Finally, we observed 5 isolates that were resistant to both erythromycin and clindamycin, and yet contained none of the common resistance genes for which we tested, which is consistent with previous reports [22, 23]. This may suggest an alternatemechanism of resistance. A plausible mechanism is mutation in the 23S rRNA. Such mutations have been reported to cause macrolide resistance in *S. pneumoniae* [24]. Further study of these isolates is warranted.

## Figures and Tables

**Table 1:** Detection of macrolide and lincosamide resistance genes in GBS. E denotes erythromycin; CC denotes clindamycin; S denotes susceptible; R denotes resistant.

Phenotype	Number of isolates	*erm*B only	*erm*TR only	*mef*A only	*erm*B and *erm*TR	*erm*B and *mef*A	*erm*TR and *mef*A	No *erm* or *mef*A

E-S, CC-S	240	9	9	13	—	2	—	207
E-R, constitutive CC-R	79	51	7	—	10	5	1	5
E-R, CC-S	—	—	—	—	—	—	—	—
D-test + (inducible CC-R)	8	—	7	—	1	—	—	—
D-test - (M phenotype)	11	3	1	4	—	2	—	1
E-S, constitutive CC-R	2	—	1	—	—	—	—	1
Total	340	63	25	17	11	9	1	214

**Table 2:** Comparison of discordant phenotype and genotype among 340 GBS isolates. E denotes erythromycin; CC denotes clindamycin; S denotes susceptible; R denotes resistant.

Phenotype	Genotype	No. of isolates

E-S, CC-S	*erm*B only	9
E-S, CC-S	*erm*TR only	9
E-S, CC-S	*mef*A only	13
E-S, CC-S	*erm*B and *mef*A	2
E-R, CC-S, D-test - (M phenotype)	*erm*B only	3
E-R, CC-S, D-test - (M phenotype)	*erm*TR only	1
E-R, CC-S, D-test - (M phenotype)	*erm*B and *mef*A	2
E-R, constitutive CC-R	No mechanism detected	5
E-S, constitutive CC-R	*erm*TR	1
E-S, constitutive CC-R	No mechanism detected	1
Total	—	46 (14%)
